# Acoustic Sensing and Ultrasonic Drug Delivery in Multimodal Theranostic Capsule Endoscopy

**DOI:** 10.3390/s17071553

**Published:** 2017-07-03

**Authors:** Fraser R. Stewart, Yongqiang Qiu, Holly S. Lay, Ian P. Newton, Benjamin F. Cox, Mohammed A. Al-Rawhani, James Beeley, Yangminghao Liu, Zhihong Huang, David R. S. Cumming, Inke Näthke, Sandy Cochran

**Affiliations:** 1 School of Life Sciences, University of Dundee, Dundee DD1 5EH, Scotland, UK; f.w.stewart@dundee.ac.uk (F.R.S.); i.z.newton@dundee.ac.uk (I.P.N.); i.s.nathke@dundee.ac.uk (I.N.); 2 School of Engineering, University of Glasgow, Glasgow G12 8QQ, Scotland, UK; Yongqiang.Qiu@glasgow.ac.uk (Y.Q.); Holly.Lay@glasgow.ac.uk (H.S.L.); Mohammed.Al-Rawhani@glasgow.ac.uk (M.A.A.-R.); James.Beeley@glasgow.ac.uk (J.B.); David.Cumming.2@glasgow.ac.uk (D.R.S.C.); 3 School of Medicine, University of Dundee, Dundee DD1 9SY, Scotland, UK; b.cox@dundee.ac.uk; 4 School of Science and Engineering, University of Dundee, Dundee DD1 4HN, Scotland, UK; y.u.liu@dundee.ac.uk (Y.L.); z.y.huang@dundee.ac.uk (Z.H.)

**Keywords:** ultrasound, capsule endoscopy, USCE, UmTDD, targeted drug delivery, theranostics, gastrointestinal, acoustic sensing, ultrasonic drug delivery, endoscopy

## Abstract

Video capsule endoscopy (VCE) is now a clinically accepted diagnostic modality in which miniaturized technology, an on-board power supply and wireless telemetry stand as technological foundations for other capsule endoscopy (CE) devices. However, VCE does not provide therapeutic functionality, and research towards therapeutic CE (TCE) has been limited. In this paper, a route towards viable TCE is proposed, based on multiple CE devices including important acoustic sensing and drug delivery components. In this approach, an initial multimodal diagnostic device with high-frequency quantitative microultrasound that complements video imaging allows surface and subsurface visualization and computer-assisted diagnosis. Using focused ultrasound (US) to mark sites of pathology with exogenous fluorescent agents permits follow-up with another device to provide therapy. This is based on an US-mediated targeted drug delivery system with fluorescence imaging guidance. An additional device may then be utilized for treatment verification and monitoring, exploiting the minimally invasive nature of CE. While such a theranostic patient pathway for gastrointestinal treatment is presently incomplete, the description in this paper of previous research and work under way to realize further components for the proposed pathway suggests it is feasible and provides a framework around which to structure further work.

## 1. Introduction

Gastrointestinal (GI) disorders represent a myriad of conditions related to known and unknown causative agents. These conditions include food-borne illnesses (gastroenteritis), inflammatory bowel disease (IBD: Crohn’s disease, ulcerative colitis) and neoplastic diseases (Barrett’s esophagus, colorectal cancer). Due to the number and common occurrence of conditions associated with the GI tract, gastroenterology is one of the most heavily utilized areas in healthcare systems. Furthermore, many of these conditions demonstrate increasing upward trends. This is particularly true for IBD which affects more than 2 million people in the USA [1] and colorectal cancer, related to ageing populations [2].

GI endoscopy is one of the most heavily used procedures in gastroenterology and hospital services in general [1]. It permits entry via natural orifices in a minimally invasive manner with access granted to the entire length of the GI tract. The upper GI tract is accessed with esophagealgastroduodenoscopy (OGD), the lower section with colonoscopy and the less routine procedure of enteroscopy allows access to the remote small bowel. All three approaches allow the clinician direct visual assessment of the mucosa and subsurface structures with endoscopic ultrasound (EUS). Furthermore, conventional endoscopes allow tissue biopsy and means of treating pathology, either pharmaceutically or physically. However, despite its obvious utility for diagnosis and treatment, conventional endoscopy places demands on hospital resources in terms of operator training, patient management and capital expense [3].

Video capsule endoscopy (VCE) [3] has been introduced and accepted as a routine clinical procedure in the past twenty years as an alternative to conventional endoscopy. Its development has benefited from three areas of innovation: camera miniaturization and related electronics; minimization of power consumption from an on-board capsule battery; and wireless telemetry for data communication with a remote data recorder. Automated information extraction and interpretation, i.e., computer aided diagnosis (CADx), is a fourth innovation that is still a topic of research. However, capsule localization and positioning accurate to a level compatible with targeted therapies remain elusive and are an area of active research. 

The conventional diagnostic modality of optical endoscopy, as noted, is now complemented by multiple additional modalities for diagnosis. The most important is ultrasound (US) imaging [4], due to its ability to visualize subsurface pathology. The adoption of endoscopic US imaging is based on the nature of US as safe, inexpensive and capable of real time imaging deployed at the point of care [5]. In addition to qualitative diagnosis, US data have been shown to be amenable to quantitative analysis and US carries the potential for therapeutic treatment through ultrasound-mediated targeted drug delivery (UmTDD). 

A means for improving diagnosis and ultimately treatment is to combine multiple diagnostic and therapeutic (i.e., theranostic) modalities. Sonopill [6,7] is an example of a multimodal capsule which aims to combine US imaging with other diagnostic modalities such as video imaging, fluorescence imaging [8] and pH sensing. In addition, therapeutic capsule endoscopy (TCE) devices are also under development, an example being SonoCAIT [9,10], a capsule endoscopy (CE) device containing UmTDD components, discussed in detail in Section 4.3.

This paper describes progress towards a proposed patient pathway utilizing multimodal CE theranostically. We first provide an in-depth review of CE for both diagnosis and therapy in Section 2, particularly highlighting the role of US. In Section 3 we describe the proposed patient pathway for a multimodal CE system in the clinic. Then in Section 4 we outline results relating to each step along this pathway, with the use of microultrasound (μUS) as a means to detect diseased regions in the GI tract, the use of capsule US for fluorescent marking of tissue, and the use of fluorescence imaging for detection. Progress towards TCE containing an UmTDD system is also discussed and the usefulness of capsule-based US to enhance therapeutic agent uptake is considered.

## 2. Capsule Endoscopy

### 2.1. Video Capsule Endoscopy for Diagnosis

The first ingestible wireless CE device was announced by Given Imaging Inc. and Dr. Swain at the Royal London Hospital, UK, in 2000 [3,11]. The capsule for diagnosis in the small bowel, initially called M2A and latterly branded as PillCam^®^ SB (Medtronic Inc., Dublin, Ireland), has diameter Ø11 mm × 26 mm length and weighs 3.7 g [11]. It consists of the following components: an optically-transparent dome, a lens with a narrow aperture, four light emitting diodes (LEDs), a complementary metal oxide semiconductor (CMOS) image sensor, two silver oxide batteries, an application-specific integrated circuit (ASIC), a radio-frequency (RF) transmitter and an antenna [11]. 

Because of physiological differences, different CE devices are required for individual sections of the GI tract, exemplified by PillCam^®^ COLON2 for colonoscopy and PillCam^®^ UGI for esophagoscopy [11,12,13,14]. The dissolvable PillCam^®^ Patency capsule has also been developed to verify adequate patency of the GI tract prior to CE, aiming to reduce the risk of capsule retention [15,16]. Besides the PillCam^®^ family, other CE devices are also commercially available for small bowel endoscopy, as listed in Table 1.

Although commercial CE devices have been established to allow inspection of the entire GI tract with minimal discomfort to patients, they still suffer from several limitations relating to locomotion control (positioning), localization and movement tracking, and power supply and power management, and have restricted imaging modalities [12,19,21,25,26]. These limitations are driving technological development of research prototypes.

Current CE devices are propelled passively through the entire GI tract by peristalsis and their position, speed and orientation cannot be controlled [12,26,27]. Passive locomotion prevents prolonged diagnosis and therapeutic interventions, unlike conventional endoscopes which permit tissue biopsy and aspiration of fluid for cytology. Many active locomotion approaches have been proposed and feasibility has been explored with prototypes, including electrically-stimulated muscle contractions [28], the use of shape memory alloys (SMA) [29] and external magnetic fields [30,31] and microrobots with leg-like [32,33,34] and earthworm-like [35,36] mechanisms.

The nature of the GI tract makes it challenging to localize capsule position precisely [37]. RF triangulation with an external sensor array to estimate the capsule travel distance based on RF signal strength has been used in commercial CE devices such as PillCam^®^ M2A. However, experimental studies have shown that the noise in the RF signal measurement can cause an average error of 37.7 mm and a maximum error of 114 mm [38,39]. Magnetic tracking algorithms have shown better accuracy, with position errors less than 10 mm in many studies [40,41,42]. However, the overall accuracy is highly dependent on the number of external sensors used [21] and it is challenging to work with an external magnetic locomotion approach at the same time [40]. Other methods have also been reported based on US time-of-flight [43], X-ray radiation [26,44] and methods using gamma scintigraphy [45]. 

Most commercial CE devices rely on silver-oxide coin batteries to provide power, these being the only coin batteries approved for clinical use [20]. They provide 3 V at 55 mAh for approximately 8–10 h, with an average power delivery of 20 mW [20,21,46]. In general, small batteries with high energy density are required to prolong the operational time and extend the functionality of CE. Wireless power transfer and energy harvesting technologies have been investigated as alternatives, including inductive coupling, microwave coupling and US technology [13,21,47,48].

Typical transit times for capsules through the small and large intestines are about 3 h and 20 h, respectively [19,20,49,50]. Depending on the frame rate of CE devices (Table 1), a large number of images or lengthy video can be generated during the procedure, requiring lengthy clinical review [18]. Hence, it is important to have software that can improve the visibility of lesions and shorten the review time without sacrificing accuracy [18,25,51]. Recent software developments have focused on CADx systems and image analysis to increase diagnostic yield and reduce inter-observer variability [25].

### 2.2. Therapeutic Capsule Endoscopy

At present, most commercial capsules are used only for GI diagnosis. Their limitations make it very difficult to extend their use to more demanding diagnostic and therapeutic procedures such as biopsy, cytology, minimally invasive surgery and targeted drug delivery, all of which are possible with conventional endoscopes. These procedures require precise localization, controlled locomotion, and real-time viewing, with remote-controlled tools and components in the TCE devices [52]. A few capsules have been developed specifically for drug absorption studies; examples are Enterion and InteliSite [14,45], and the Intelligent Pill system (iPill, Koninklijke Philips N.V., Amsterdam, The Netherlands) was developed for controlled release of medication in the GI tract [53]. Colak et al. proposed a theranostic capsule to deal with obscure GI bleeding [54]. However, these capsules have only basic on-board electronics and a drug reservoir, without any imaging capability. Therefore, further research and development is required for the evolution of CE from diagnosis to multimodal theranostic robotic systems [11]. 

An effective targeted drug delivery capsule should contain anchor and release mechanisms. The anchor mechanism positions the capsules at the target site in the desired orientation. It usually deploys leg-like mechanisms [32,33,34,40,50,55] or uses a magnetic field [56,57] to attach the capsule to the inner wall of the GI tract. The release mechanism is triggered to deliver drugs in a controlled manner and involves a reservoir [12,40,58] with release triggered by specific environmental conditions, e.g., temperature or pH [50], or by activation of a magnetic field [56].

The ability to perform minimally invasive surgery is another modality desirable for TCE [12,37,59]. Valdastri et al. reported the first successful in vivo surgical experiment using wireless CE [59]. Their device, Ø12.8 × length 33.5 mm, was equipped with four permanent magnets for active external magnetic steering. A nitinol clip was mounted on the tip of the capsule for release in response to an external signal [59]. The feasibility of tissue biopsy has also been demonstrated with capsules using a rotational razor mechanism, micro-spikes or two cylindrical razors [60,61,62]. To enable identification of sites of pathology with TCE, optical biopsy has been proposed for tissue diagnosis in vivo, including fluorescence endoscopy, optical coherence tomography, confocal microendoscopy, light-scattering spectroscopy, Raman spectroscopy, and molecular imaging [20,63].

### 2.3. Ultrasound Capsule Endoscopy

Currently, all commercial diagnostic CE relies on optical images and videos to aid diagnosis. However, optical imaging is limited to the internal luminal surface of the GI tract. The addition of US imaging in CE can expand the capability to image and analyze subsurface features to resemble EUS [64]. Because in this case, US is transmitted from inside the body cavity, attenuation is reduced and a higher US frequency can be used to deliver higher resolution data than those obtained from transcutaneous US. High-resolution imaging and related quantitative analysis provided by μUS have the potential for early detection of GI disease, prior to optical manifestation. 

In 2004, Olympus announced the development of US capsules [65] but no further information has emerged subsequently. However, progress by several research groups working with prototypes [63,66,67,68,69] and several patents have been published [26,70,71,72]. Yuan et al. developed and fabricated a photoacoustic imaging endoscope with an acousto-optical coaxial structure for cavity imaging [66]. The tethered device consists of a Plexiglas tube, an optical fiber, a tapered reflector, a 64-element ring transducer array and a coupling medium [66]. Photoacoustic images of porcine colorectal tissue embedded in a transparent gelatin phantom were reconstructed to demonstrate feasibility. A European Commission project, TROY, engaged in the development of an US capsule based on a 5 MHz 32-element ring array [67]. Memon et al. reported on a capsule US device with a 128-element cylindrically-shaped capacitive micromachined ultrasonic transducer (CMUT) array [68] and Wang et al. recently reported a successful US capsule based on mechanical rotation of a 39 MHz US transducer [73]. A rotary solenoid-coil motor was employed to rotate the US transducer with sectional electronic control. Phantoms and ex vivo porcine small intestine specimens were used for image evaluation [73]. A similar concept was studied by Lee et al. [69]. 

In addition to imaging, US can also be used for therapy, including tissue ablation and site-specific drug delivery. For tissue ablation, high-intensity focused US is required. This is difficult to integrate into capsule form because of the energy requirement and size limitations and also because of the high risk of perforation of the bowel. However, UmTDD can direct liposome entrapped gas bubbles and drugs [74,75,76,77] and can be integrated into capsules [9,10]. Drug-carriers can be affected by focused US and release their contents in confined regions, i.e., the focal zone of a focused US transducer. The rate of drug release strongly depends on and thus can be modulated by US parameters such as frequency, intensity, focal size, and inter-pulse intervals [78]. With appropriate control, drug delivery can be achieved in an effective manner.

## 3. Proposed Theranostic Patient Pathway

The important components for multimodal CE for diagnosis are actuation and sensing devices, a power supply, wireless communication control circuitry and an antenna. In addition, TCE devices must contain a targeting mechanisms and a drug reservoir and release mechanism. The typical shape of a clinical CE device is a cylinder with hemispherical ends, Ø_CE_ = 10 mm and overall length L = 30 mm [79]. Devices described above correspond closely to these norms. Due to the complexity of the devices and their relatively small size, a combination of two or more theranostic devices in succession may be necessary to deliver the required payload. Such a multi-capsule approach has the extra advantage that clinical validation can be included in the diagnostic procedure to allow a second opinion before administration of therapeutics.

**Figure 1:**
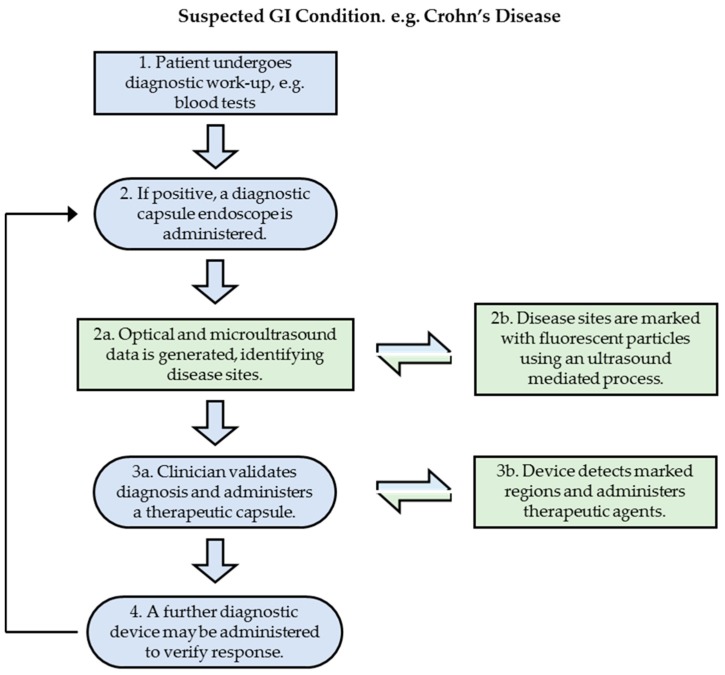
Schematic of the proposed patient pathway.

The proposed patient pathway (Figure 1) begins with a patient presenting with symptoms that indicate a GI condition, for example Crohn’s disease. In the next step, the patient is subjected to conventional diagnostic procedures and, if positive, a diagnostic CE device is administered that uses white light optics and μUS imaging to identify diseased regions in the GI tract after video review and quantitative US (QUS) analysis. The diseased regions are marked with fluorescent nanoparticles using a process analogous to UmTDD. At this stage, a clinician validates the diagnosis and can then apply TCE. The therapeutic device is designed to detect the fluorescent nanoparticle markers without the need for further diagnostic or communications functions, allowing space in the capsule for a reservoir of therapeutic agents, a release mechanism and US components to direct the agents towards the treatment site while simultaneously assisting in separating the active component of the agent, i.e., a drug, from a carrier, e.g., a microbubble, and increasing tissue permeability for enhanced uptake. Following delivery, further diagnostic CE could be used to assess therapeutic efficacy. Table 2 links the proposed patient pathway to current and future status of research described in this paper with relevant references highlighted.

## 4. Results

The proposed theranostic patient pathway described above is a complex procedure. However preliminary results have been achieved for many of the individual steps, as described in this section and in the literature as highlighted in Table 2. Together, these highlight the minimally invasive nature and strong reliance on contemporary sensing techniques of USCE.

### 4.1. Microultrasound Diagnosis and Quantitative Analysis

Maximum efficacy of ultrasonic drug delivery requires accurate identification of the nature and extent of the diseased tissue to be treated. This can be achieved through the appropriate use of qualitative and quantitative µUS imaging. Previous research has shown good agreement between µUS and histology when imaging human GI tissue [85]. We seek to expand on this work through the use of quantitative analysis techniques to calculate key tissue properties and establish a healthy baseline. Quantitative µUS can detect pre-cancerous changes in tissue organization in isolated tissue in a laboratory environment [86,87,88,89]. Both acoustic impedance (Z) and backscattering coefficient (BSC) showed potential for detecting changes in cell and tissue architecture characteristic of early disease [86]. 

In new studies we used a 48 MHz piezocomposite transducer (AFM Ltd., Birmingham, UK) with a mechanical scanner [80] to obtain µUS images of ex vivo porcine tissue samples and processed them digitally with QUS techniques. To establish a measurement methodology compatible with clinical systems, reference scans of tissue separated into its component layers (homogeneous tissue) were used in conjunction with an automated tissue segmentation algorithm for characterization of tissue in situ without the need for physical biopsy.

Healthy ex vivo porcine tissue from the esophagus and small bowel were used to model the human GI tract [90]. Post mortem tissue was obtained fresh-frozen from the abattoir so it was not necessary to obtain ethical approval. While the initially frozen state of the tissue was a consideration, freeze-thaw cycles have been shown not to affect acoustic properties [91]. The esophageal tissue can be separated into four layers: (1) mucosa, (2) submucosa, (3) muscularis propria and (4) adventitia and serosa. As precancerous tissue disruption is commonly found in the mucosa and submucosa [92], this work focused on those layers.

#### 4.1.1. Tissue Characterization Techniques

A critical challenge in translating laboratory analysis techniques to clinical applications is the loss of known, fixed reference points. Established protocols for QUS commonly use flat substrates and samples with well-controlled, known thicknesses to minimize data variance [93,94]. By contrast, in vivo approaches commonly use approximations which eliminate those reference signals [88,95]. For the present demonstration, a hybrid approach was used, in which reference values obtained with homogeneous, healthy samples were combined with digital image segmentation to calculate the desired values in heterogeneous tissue, specifically to identify Z, BSC and attenuation.

For two well-differentiated materials, the percentage of an incident US wave reflected from the interface between them is approximated as a function of their acoustic impedances [81]. Based on this, the acoustic impedance of an unknown tissue can be calculated from the impedance of the imaging medium (*Z_w_*), the incident pressure wave (*V_i_*) and the reflected wave (*V_r_*), giving *Z_t_* (MRayl):

(1) $$Z_{t}=\;-\frac{V_{r}+V_{i}}{V_{r}-V_{i}}Z_{w},$$

The incident wave amplitude can be obtained by reversing the calculation using the same transducer configuration and a material of known acoustic impedance as a reflector, in this case a quartz flat. The BSC was calculated using the same methodology as in previous studies [86], where the coefficient is broken down into terms based on the geometry of the transducer, a reference echo from a known quartz reflector and loss due to attenuation:

(2) $$\mu_{B}=\frac{R_{q}}{2\pi \left(1-\cos\theta_{T}\right)}\frac{\int_{t_{1}}^{t_{2}}{\left|V_{s}\left(t\right)\right|}^{2}dt}{\int_{-\infty }^{\infty }{\left|V_{q}\right|}^{2}dt}\frac{4a^{\prime }}{e^{-4a^{\prime }d_{1}}-e^{-4a^{\prime }d_{2}}}$$

where *R_q_* is the reflection coefficient of the quartz reflector, *θ_T_* is the half-angle subtended by the transducer face at the focal point, *V_s_* is the signal obtained from the interrogated sample, integrated over the time-gate of interest, *V_q_* is the amplitude of the signal obtained from the ground truth reference integrated over the full sample time, and *d*_1_ and *d*_2_ are the minimum and maximum depths of the tissue sample within the US image.

Before the BSC could be calculated for a given sample, the loss of ultrasonic signal due to tissue attenuation had to be determined, requiring an accurate measurement of both the sample thickness and the specific attenuation for the tissue. Methods exist for calculating attenuation in vivo [96] but they require significant additional processing and are not as accurate as ex vivo methods using known reference reflectors. For this reason, the attenuation here was pre-calculated using manually separated samples and these values were applied when calculating the BSC in vivo.

For a tissue sample of thickness *d*, the attenuation factor, *α*, (dB mm^−1^) can be calculated as:

(3) $$\alpha =\frac{-20}{2\times d}\log_{10}\frac{V_{r}}{V_{a}},$$

where *V_r_* is the amplitude of the signal from the reference reflector beneath tissue and *V_a_* is the amplitude of the signal from the reference reflector without tissue present. The logarithmic attenuation coefficient (dB mm^−1^) can then be converted to the natural logarithmic *a*′ (neper mm^−1^):

(4) $$a^{\prime }=\frac{\alpha }{8.686},$$

#### 4.1.2. Experimental Tissue Preparation

All porcine tissue samples were sourced from an abattoir (Medical Meat Supplies Ltd., Oldham, UK) and were supplied frozen, certified fit for human consumption, and thus deemed suitable to provide a healthy baseline. Esophageal samples were selected from the transition from the esophagus to the stomach through the gastro-oesophageal junction (GOJ) which provides naturally heterogeneous tissue for qualitative and quantitative assessment. Small bowel samples were obtained with the mesenteric vessels intact so the samples could be perfused to ensure accurate mucosal surfaces for drug delivery trials.

Prior to scanning, samples were thawed in their vacuum packs for 20 min in water at room temperature and then rinsed with water after removal from the vacuum pack. Samples were then transferred to a tray containing 2 cm thick acoustic absorber covered by ~4 cm of 1% agar by mass. Trays were prepared 1 day prior to scanning to allow the agar substrate to solidify overnight at room temperature. Samples were coupled to the agar using standard acoustic gel and covered with degassed phosphate buffered saline (dPBS) to prevent tissue degradation during scanning and ensure ultrasonic coupling to the ultrasound devices.

The scanning apparatus used in the imaging experiments was configured for planar tissue samples so the esophageal samples were prepared for scanning by bisecting them along the long axis using a scalpel. This allowed the tissue to be opened and pinned to the agar without disrupting the GOJ. To obtain homogeneous samples of the mucosa and submucosa for attenuation measurement, two esophageal samples were prepared using the same method as that used for the whole samples but they were then separated along the fascial plane of the tissue with a scalpel. The mucosa/submucosa combination was separated from the remaining layers, then mounted on the agar substrate for imaging.

#### 4.1.3. Results

**Figure 2:**
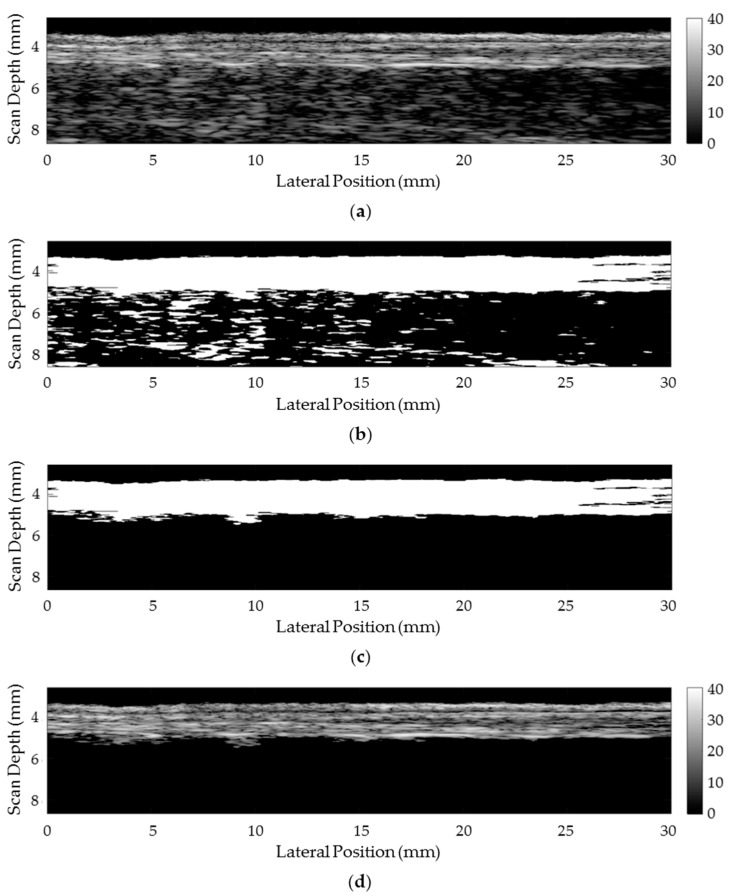
Sequencing for the digital segmentation of the heterogeneous tissue samples to isolate the mucosa/submucosa. All images are shown with the superficial layer towards the top of the image. (**a**) Original ultrasound image (**b**) Initial threshold mask (**c**) Processed mask (**d**) segmented image.

A key component in successful characterization of the mucosa/submucosa in vivo using the approach proposed here is the ability to separate the region of interest (ROI) digitally from the surrounding tissue in the µUS scans. A custom software algorithm was developed in MATLAB (The Mathworks, Cambridge, UK) to isolate the data from the ROI for subsequent processing. This algorithm takes the original data from each B-scan, Figure 2a, and separates it into echoes with amplitudes above and below an empirical threshold of −26 dB with respect to the brightest echo. The resulting binary mask, Figure 2b, is cleaned up by applying a closing operation and the largest echo region is selected as the ROI, Figure 2c. The mask is then applied to the original data to remove the echo data outside the ROI, Figure 2d. BSC and Z are then calculated for this segmented data.

**Figure 3:**
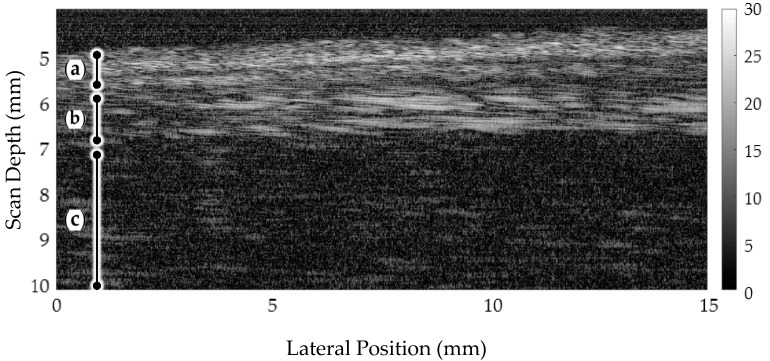
48 MHz microultrasound scan of ex vivo porcine esophageal tissue. Tissues layers can be distinguished as (**a**) mucosa, (**b**) submucosa and (**c**) muscularis propria and serosa.

When imaging the tissue for each sample, three B-scans were obtained along the long axis, spaced 0.5 mm apart. The images were then inspected and any samples with non-identifiable irregularities were removed from the sample set. The resulting images, an exemplar being shown in Figure 3, were assessed by a clinician who was satisfied with the layer differentiation obtained.

The attenuation coefficient for each vertical line in the images of the two mechanically separated samples was calculated from Equation (3), then the values were averaged across both samples to obtain *α_av_* = 1.86 ± 0.72 dB mm^−1^. This is on the same order as the attenuation seen in other low density tissues [97] similar to mucosa/submucosa and this average value was used in all subsequent calculations.

**Figure 4:**
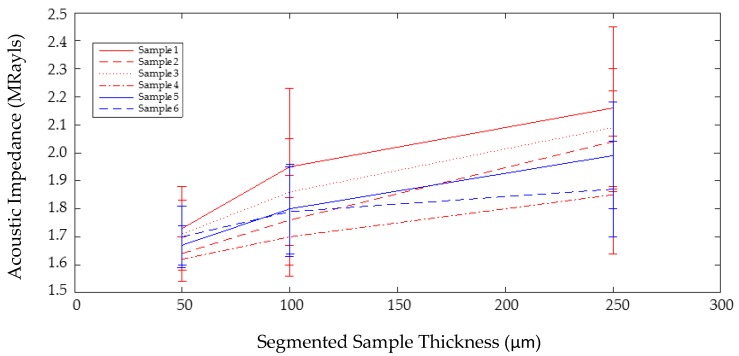
Increase in acoustic impedance as a function of segmented tissue thickness. Error bars show standard deviation across the full 30 mm scan for each sample.

**Figure 5:**
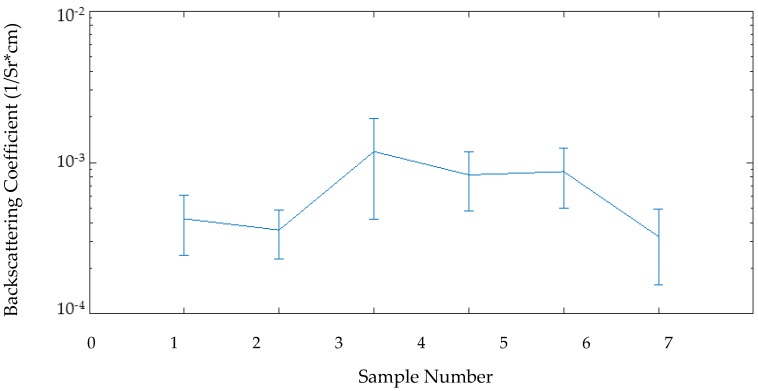
Backscattering coefficients for each digitally segmented tissue sample as a function of sample. Error bars show standard deviation across the full 30 mm of each scan.

For all samples, Z and BSC were calculated for individual vertical lines then the average and standard deviation for all lines was calculated for each sample. The variation in Z across the six samples and the different segmentation depths can be seen in Figure 4 and the BSC intersample variance is shown in Figure 5. 

Clinical analysis of the µUS images of the porcine GI tissue determined that diagnostic quality layer differentiation was achieved with the qualitative images with the clinician able to determine thickness and variability of the different layers of the tissue to his satisfaction. 

The attenuation values measured in the reference sample showed higher than ideal variability across the samples but the values obtained were within the expected range. Future work will focus on error reduction through better thickness control of the reference samples.

The segmentation algorithm successfully isolated the mucosa/submucosa digitally from the other tissue layers using a single threshold value to allow automated ROI detection in real-time, making this a feasible approach for in vivo scanning. It also allowed variable thickness segmentation, as demonstrated in the acoustic impedance analysis, revealing an increase in apparent impedance with segmentation thickness. This may correlate with the increase in density expected in healthy tissue [86]. Further work is required to determine if disruption in the macrostructure of the tissue, as seen in pre-cancerous sample, would alter this trend. Some variance was seen in the BSC values across the six samples but this is also reported in the literature [93] suggesting that BSC measurement is prone to biological variability.

### 4.2. Fluorescent Nanoparticle Marking and Imaging

Following identification of diseased regions in the proposed patient pathway, these regions must be marked with fluorescent nanoparticles in an US-mediated process. This is necessary, assuming that it is impossible to include both full diagnostic capabilities and the components required for therapy in a single CE device of viable dimensions. This section briefly describes the process of marking tissue with fluorescent nanoparticles and explains the fluorescent imaging that could be used to detect the marked regions.

The marking process was demonstrated by Cox et al. [82]. Experiments were performed on ex vivo small bowel tissue taken from wild type mice using fluorescent CdSeS/ZnS quantum dots (QDs) (Sigma-Aldrich Corp., St. Louis, MO, USA) that were directed towards the focus of a miniature US transducer. The design of this transducer [84] was identical to those used in TCE research [9,10,83] but they had different casings and connectors. The transducer was driven with an excitation voltage of 10 *V_pp_* for *t* = 6 min with the QDs introduced at time *t* = 5 min for a total time of 1 min using a Braun syringe driver (Braun GmbH, Kronberg, Germany). Post sonication, tissue was washed twice with phosphate buffered saline (Thermo Fisher Scientific, Waltham, MA, USA) and illuminated using an ultraviolet fluorescent lamp (UVGL-58, Analytik Jena, Jena, Germany). 

The fluorescence imaging results showed an increased concentration of QDs where tissue was insonated. The precise location of the QDs, in the tissue or only within the mucus layer, has not yet been determined but both locations may be suitable as markers of diseased tissue as discussed in the proposed patient pathway. Further clinically-based research is still needed to determine the lifetime of the marker and its stability at the insonated location.

**Figure 6:**
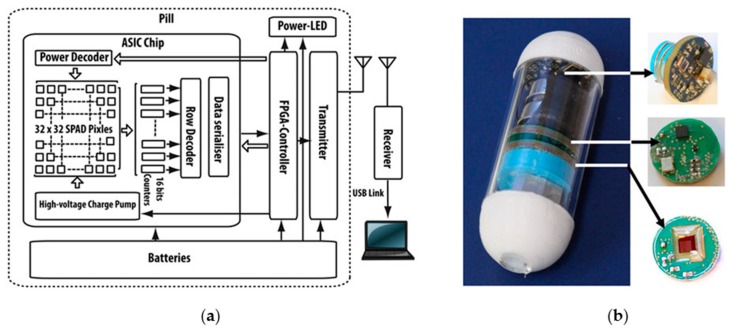
(**a**) Block diagram of fluorescence-imaging capsule and (**b**) final manufactured test capsule, Ø 16 mm. [8].

To mark tissue with fluorescent agents using US transducers that have excitation amplitudes appropriate for CE implementation also requires a means to visualize the marked region in vivo. Here, the work of Al-Rawhani et al. [8] is notable. They used fluorescence imaging with a single-photon avalanche diode (SPAD) array in application-specific integrated circuits (ASIC) housed in a CE device (Figure 6). The authors demonstrated successful miniaturization and imaging, putting in place a key component in the patient pathway proposed here. It is also possible that the same technique could be used during original diagnosis, as a complementary or alternative method to the combination of white light and US imaging discussed here. Indeed, the multimodal approach when used as a complementary method may be highly attractive in some clinical situations.

### 4.3. Feasibility of Therapeutic Capsule Endoscopy

As discussed in Section 2.2, there has been little progress towards the development of TCE. Previous therapeutic capsule designs have had limited success such as the inability to deliver therapeutic agents through the mucosa, poor localization and positioning. One solution is to capitalize on UmTDD to overcome these problems. UmTDD can facilitate delivery of therapeutic agents at the target location. Focused US can release therapeutic agents from their carrier and increase uptake into cells through thermal and mechanical effects. Although UmTDD has not yet achieved clinical use, there are systems under investigation that are designed for extracorporeal application. These require relatively high-power levels and are sometimes combined with magnetic resonance imaging.

A specific challenge in realizing UmTDD components in CE form is the necessary miniaturization of the focused-US transducer. A CE US transducer is much smaller than a conventional UmTDD transducer, until recently it has been unclear whether it can produce enhance drug uptake. Following the previous descriptions of key issues in a patient pathway for TCE, this section of the paper describes the development of a proof of concept TCE device, named SonoCAIT. Prototype capsules are tethered for power and drug delivery. Nonetheless, they can provide the proof of concept that UmTDD components can fit within the volume of a capsule and can establish the effectiveness of miniature US transducers.

#### Capsule Fabrication and Functional Testing

Components necessary for a proof-of-concept UmTDD capsule are: a miniature focused-US transducer, video camera with illumination, therapeutic agent channel, capsule shell and tether. 

**Figure 7:**
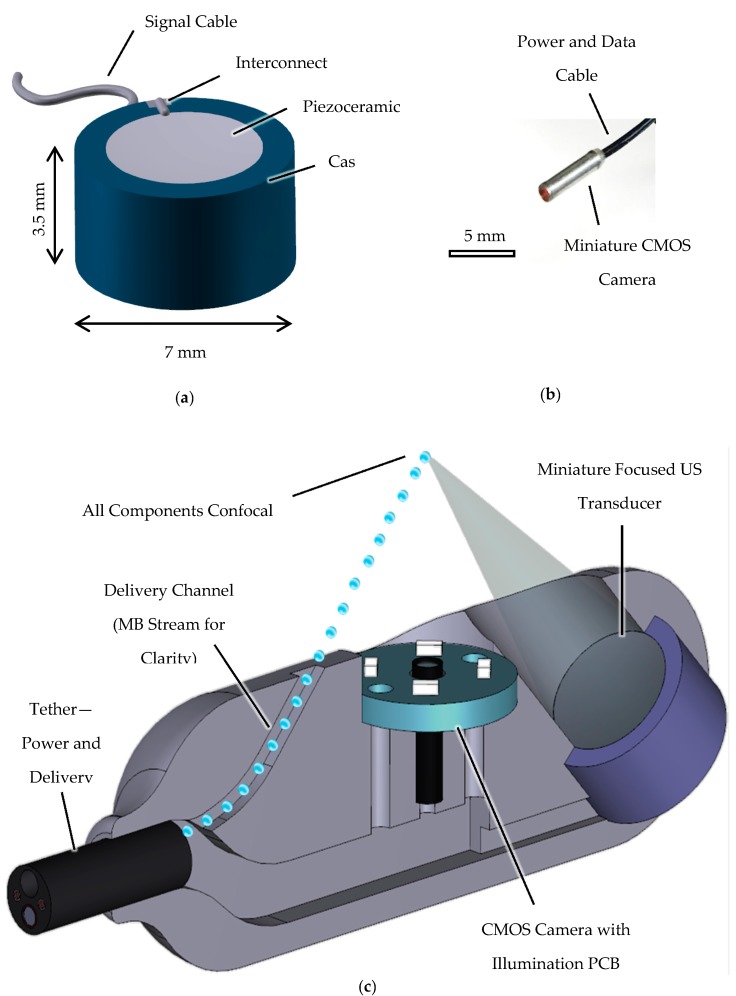
(**a**) CAD cross section of the miniature focused-US transducer with PZ54 bowl, backing layer, interconnects and casing. (**b**) Micro ScoutCam imaging camera 1.2 mm in diameter, 5 mm in length, tethered. Camera passes through illumination board with four LEDs mounted on a PCB with diameter 8 mm. (**c**) CAD cross section of the capsule with components included. All components are confocal and the US beam and therapeutic agents are indicated in the drawing. The capsule has diameter *D_cap_* = 10 mm and length *L_cap_* = 30 mm.

A typical focused-US transducer consists of a piezoelectric element, backing layer for physical support with minimal energy absorption, electrical interconnects and a protective casing. The piezoelectric element chosen for the present application was a PZ54 piezoceramic bowl (Meggitt Sensing Systems, Kvistgaard, Denmark) with outer diameter, OD = 5 mm, radius of curvature, *R_c_* = 15 mm, thickness, *T* = 0.5 mm, and central driving frequency, *f_c_* = 4 MHz. PZ54 was chosen was developed specifically for focused US applications [98] The backing layer consists of a 3:1 mass ratio mixture of K1 glass microbubbles (3M, Maplewood, MN, USA) and epoxy (Epofix, Struers A/S, Denmark). The Ag electrodes fired onto the PZ54 bowl were connected to a coaxial cable with OD = 0.3 mm and ID = 0.1 mm using conductive silver epoxy (G3349, Agar Scientific, UK). The transducer is housed in a casing with diameter, *D_case_* = 7 mm, and length, *L_case_* = 3.5 mm. The casing was printed in ABS plastic using a Replicator 3D printer (MakerBot, New York, NY, USA). The entire fabrication process is detailed elsewhere [9] and the fully fabricated transducer is shown in Figure 7a.

The camera used in the present work (microScoutCam, Medigus Ltd., Israel) measures 1.2 mm in diameter and 5 mm in length, allowing it to fit within a capsule. The camera has an image area of 492.8 µm × 488.4 µm with a resolution of 220 × 224 pixels. Images and video are captured by a dedicated video processor to which the camera is connected. Illumination was provided by mounting four 40 mW LEDs (OSRAM Opto Semiconductors GmbH, Germany) on a printed circuit board (PCB) with a central hole, diameter 1.5 mm, to allow the camera to pass through. The camera is shown in Figure 7b.

To simplify the drug delivery mechanism for the proof-of-concept capsule, a fine bore polythene tube runs the length of the tether and into the capsule, with OD = 0.96 mm, ID = 0.58 mm; therapeutic agents are delivered through it using a syringe pump located at the distal end. The multi-channel tether has OD = 2.25 mm, ID = 1.65 mm, and connects capsule components with benchtop apparatus. It is designed such that any rotation at the proximal end corresponds to the same rotation at the distal end to assist in positioning the capsule relative to the target location.

The capsule shell was designed such that the focused-US transducer, delivery channel, video camera and illumination are all confocal to allow therapeutic agents to be released from their carriers in close proximity to the bowel wall. This will occur near the US focus, aiding the release of the agents and increasing tissue permeability. Figure 7c is a computer aided design (CAD) drawing (SolidWorks, Dassault Systèmes SOLIDWORKS Corp. Waltham, MA, USA) of the capsule and components. The capsule shell was manufactured using an Object Connex 500 3D printer (Stratasys Ltd., Minnesota, MN, USA).

For a simple demonstration of the capabilities of the components within the TCE device, glass microbubbles (MBs) (3M, Maplewood, MN, USA) were passed through the delivery channel and into the US focus, while monitoring the confocal zone with the camera. The original trajectory of the MB stream was deflected by an angle greater than 90° when it impinged on the US beam driven with an excitation voltage of 8 *V_pp_* [2]. This shows that the miniature US transducer can direct a simple agent towards a target location under visual observation. 

For the patient pathway, we propose to treat localized diseases in the GI tract using drugs in a targeted manner. UmTDD can help via three mechanisms [83]: drugs can be packaged to minimize systemic effects during delivery and can be released in the US focal zone; the drug can be directed towards the target; and US can increase permeability of the treatment site and enhance uptake. One approach illustrates the first mechanism [75] is to combine a drug with a chemically engineered package. An example was demonstrated by producing a chemical complex with doxorubicin (DOX), a chemotherapeutic drug, and γ-cyclodextrin. Comparing the effect of the complex to DOX alone showed that the complex had a reduced effect on cells in vitro but that its effect was enhanced with mild hyperthermia and cavitation caused by focused-US [75]. An approach for the second mechanism was described in Section 4.3, and demonstrated that the trajectory of MBs can be deflected with a miniature focused-US capsule transducers. The third mechanism will be investigated in this section, which explores the permeabilization effects of miniature focused-US transducers on relevant epithelial cell models. An automated cell insonation system was created to ensure reproducible experiments, miniature focused-US transducers were produced and used with the system that match those used in the TCE device, and finally experiments were preformed using an epithelial cell model.

### 4.4. Ultrasound-Mediated Targeted Drug Delivery

#### 4.4.1. Epithelial Cell Model of the Small Intestine

To investigate US facilitated permeabilization and improved uptake, requires an appropriate cell model of the GI tract. One such model are human epithelial colorectal adenocarcinoma (Caco-2) cells. Once Caco-2 cells have become differentiated and polarized, they mimic the enterocytes lining the small intestine, forming cellular junctions and microvilli [99]. They act as a model of the small intestine and are approved by the US Food and Drug Administration (FDA) [100]. Typically, it takes Caco-2 cells 21–25 days growing on ThinCert membranes (Greiner Bio-One, Kremsmunster, Austria) to form this fully differentiated cell layer. 

We maintained Caco-2 cells in Dulbecco’s Modified Eagle’s Medium (ThermoFisher Scientific, Waltham, MA, USA), supplemented with: 10% fetal bovine serum (GE Life Sciences, Chicago, IL, USA); 1% non-essential amino acids (Gibco, ThermoFisher Scientific, Waltham, MA, USA); 0.5% penicillin streptomycin (Gibco, ThermoFisher Scientific, Waltham, MA, USA). They were seeded at a density of 500,000 cells per 12-well ThinCert membrane and media was replenished daily. Transepithelial electrical resistance (TER) was measured every third day to assess barrier function using a Millicell-ERS TER meter (Millipore, Billerica, MA, USA) to measure resistance and Equation (5) to calculate TER:

(5) $$TER=\left(R_{es}-Res_{control}\right)\times Area_{ThinCert}$$

where *Res* is the direct meter reading, *Res_control_* is the resistance measured across a blank ThinCert and *Area_ThinCert_* is the area of a ThinCert. TER values in the range 500–1000 Ω·cm^2^ reflect acceptable values to represent a model of the small intestine and were usually established after 21–25 days [101,102].

#### 4.4.2. Transducers for Insonation

To study the effects of the miniature focused-US transducers on Caco-2 cells an insonation system was developed [84] utilizing transducers matching those in the TCE device. However, this system had drawbacks including an inability to insonate more than one sample at a time and that it could work only with therapeutic agents mixed in suspension not introduced through a channel as they are in the capsule. To overcome these challenges, an enhanced system was developed that could automatically insonate chosen wells in a ThinCert plate. Miniature focused-US transducers were also developed for the system, similar to those used in the capsule except for a central hole through which therapeutic agents can be passed. 

For these transducers, the piezoceramic components were PZ26 (Meggitt Sensing Systems, Kvistgaard, Denmark) perforated spherical-section bowls with OD = 5 mm, 1 mm central hole, radius of curvature, *R_c_* = 15 mm, and operating frequency, *f* = 4 MHz. The central hole allows a delivery channel to be integrated into the transducer for the introduction of therapeutic agents, similar to the setup described in Section 4.3. The PZ26 bowl is housed in a protective and structurally supporting case produced by additive manufacturing of VeroBlack material using an Object Connex printer (Stratasys Ltd., Minnesota, MN, USA). The backing layer comprised a 1:3 mass ratio mixture of K1 glass microbubbles (3M, Maplewood, MN, USA) and epoxy (Epofix, Struers A/S, Denmark). The MB-loaded epoxy was applied to the rear surface of the PZ26 material and both components were placed in a 70 °C oven to cure for 15 min. The delivery channel consisted of fine bore polythene tubing, OD = 0.96 mm, ID = 0.58 mm. The backing layer was penetrated by a 1 mm drill bit and the tubing was passed through the center of the PZ26 material, through an outlet in the edge of the casing. 

**Figure 8:**
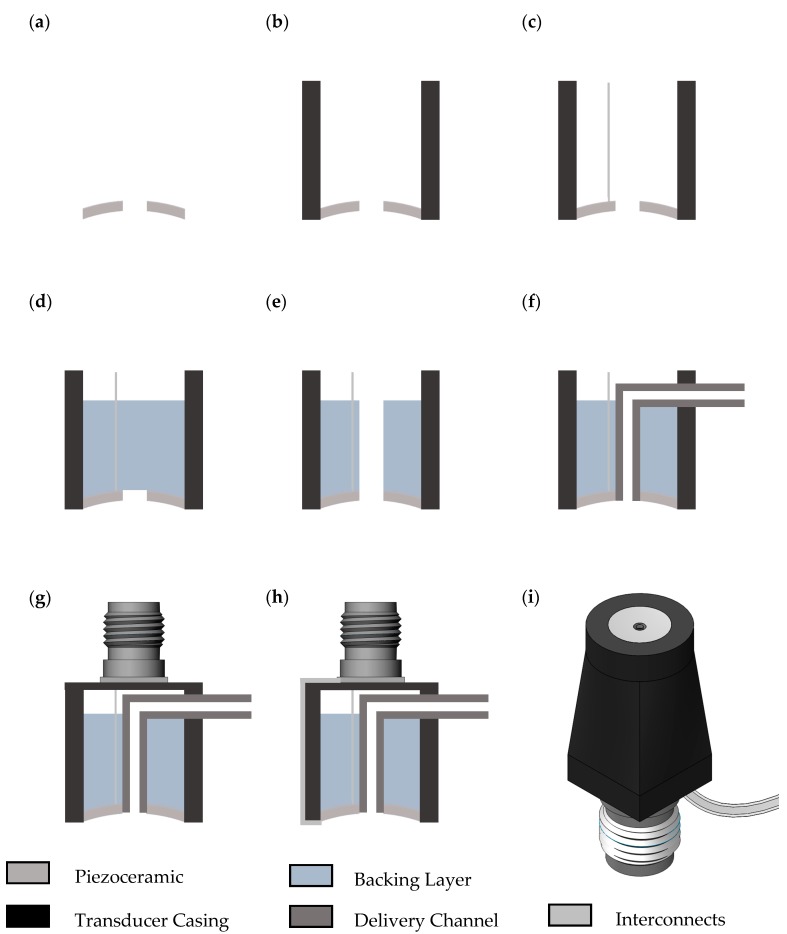
(**a**) Piezoceramic material was placed face down on a flat glass surface. (**b**) An additively-manufactured casing was placed over the piezoceramic material, ensuring they were coaxial. (**c**) Electrical interconnect was attached to the rear surface of the piezoceramic using Ag-loaded epoxy. (**d**) Glass microbubble-loaded epoxy was added to the rear surface of the piezoceramic and cured in an oven for 15 min at 70 °C. (**e**) A hole was drilled through the backing layer a 1 mm dia. drill bit. (**f**) The delivery channel was run through the central hole in the piezoceramic and out an outlet in the side of the casing. (**g**) The central pin on the surface mount SMA connector was attached to the electrical interconnect on the rear surface of the transducer using Ag-loaded epoxy. The SMA connector fit into grooves in the additively-manufactured casing and was secured using epoxy. (**h**) The electrical ground connection was attached from the outside of the SMA connector to the front face of the piezoceramic material using Ag-loaded epoxy. The ground connection runs along the outside of the transducer casing. Gaps in the transducer were sealed with epoxy to waterproof the transducer. (**i**) CAD model of the fully fabricated transducer showing piezoceramic, delivery channel, casing and SMA connector.

The silver electrodes fired onto the PZ26 bowl were connected to coaxial cable, OD = 0.3 mm, using conductive Ag-loaded epoxy (G3349, Agar Scientific, UK) and the other end of this cable was connected to the central pin of a surface mount SMA connector using conductive Ag-loaded epoxy. The SMA connector was then inserted into grooves in the additively-manufactured casing, allowing easy interchangeability of transducers in the insonation system. The fabrication process is shown in Figure 8a–h and the fully fabricated transducer CAD is shown in Figure 8i.

Acoustic power, *P_ac_*, measurements are a primary feature of transducer characterization and provide an important quantitative description of output. *P_ac_* is measured by a radiation force balance (RFB), providing quantification for both diagnostic and therapeutic US. A RFB consists of an absorbing target suspended in a degassed water bath attached to a precision balance. The displacement of the target’s equilibrium position when insonated is detected by the precision balance and the magnitude of the equivalent mass is related to *P_ac_*. In the present work, a transducer under test was mounted in a bath of degassed water with the absorbing target of the RFB (Precision Acoustics, Dorchester, UK) at the transducer focus. The transducer was driven by an Agilent 33220A signal generator (Keysight Technologies, Santa Rosa, CA, USA). Raw data was converted into *P_ac_* values using an appropriate temperature-specific calibration factor supplied by the manufacturer. The transducer was driven with input voltages in the range 3–10 *V_pp_* at 1 *V_pp_* increments. *P_ac_* was calculated for each input voltage and is shown in Table 3. In general, the transducer produced *P_ac_* in the range 8.5–153 mW, corresponding to input voltages 3–10 *V_pp_*. The electroacoustic efficiency of the transducers was calculated as the ratio of the acoustic output power, *P_ac_*, and the electrical input power, *W_input_*, obtained from Equation (6):

(6) Winput= (Vpp22)2Z

where *Z* is the electrical impedance magnitude at the input frequency and *V_pp_* is the input voltage. Efficiency values displayed in Table 3 are in the range 37.2–60.3% with an average efficiency of 54%. The values of the lowest efficiencies of 37.2% and 49.0%, corresponding to input voltages of 3 *V_pp_* and 4 *V_pp_*, are attributed to the limited accuracy of the RFB when measuring low acoustic powers.

Spatial distribution of the US field in the focal region was obtained by pressure mapping carried out in an US scanning tank (Precision Acoustics, Dorchester, UK) with a 0.075 mm diameter needle hydrophone (Precision Acoustics, Dorchester, UK) attached to a three-axis motorized stage that moved the hydrophone through the US field. A LabVIEW-based program (National Instruments, Newbury, UK) is used to control the system and position the hydrophone within the tank. The transducer was first placed in the scanning tank with the hydrophone close to its focus. The precise focus in the z-axis was then located by manually moving the z-stage, axial to the transducer, until the maximum signal was found. Subsequently, the system software scans a plane in the x-y axis to find the other focus coordinates automatically and records them. 

Once the US focus had been found, an x-y planar scan was performed over an area of 5 × 5 mm with 0.075 mm step size. The waveform was applied to the transducer using an Agilent 33,220A signal generator (Keysight Technologies, Santa Rosa, CA, USA) at *f* = 4 MHz and the input voltage applied in the range 1–10 *V_pp_* in 1 *V_pp_* increments. Results are shown in Table 3, with the acoustic output pressure in the range 11–153.4 kPa corresponding to input voltages of 1–10 *V_pp_*. The beam diameter was also calculated to be 2.7 mm at −6 dB for all input voltages. Intensity and mechanical index were calculated using *P_ac_*, pressure and beam diameter and are shown in Table 3.

#### 4.4.3. Fully Automated Insonation System

**Figure 9:**
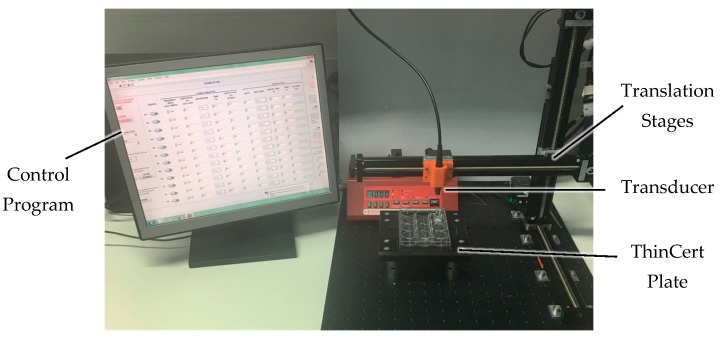
Insonation system comprising three axis translation stages, syringe pump, signal generator (off screen), cell plate holder, control program, and miniature focused US transducer.

The automated system was constructed to select one or more wells in turn, automatically lower the transducer into them, insonate the cells for a chosen time/US intensity, deliver any therapeutic agents or microbubbles, remove the transducer from the well, and move to the next well. This minimizes the human effort that was required with the previous system and allows multiple samples to be sonicated automatically [84]. The system shown in Figure 9 comprises three X-slide translation stages (Velmex Inc., New York, NY, USA) with travel length, *L_T_* = 350 mm. The stages were mounted in an x-y-z configuration to provide three degrees of freedom for accessing the wells in the plate. They were mounted onto a 450 × 600 mm optical breadboard with M6 tapped holes (Thorlabs Inc., Newton, NJ, USA) using optical cleats (Velmex Inc., New York, NY, USA). The transducer in use attached to an SMA-female to BNC-male connector that is held in an additively manufactured bracket (MakerBot, New York, NY, USA), attached to the stage carriage. A DG4102 waveform generator (RIGOL Technologies, Beijing, China) was used to drive the transducers. The therapeutic agent delivery channel was controlled by an NE-1000 syringe pump (New Era Pump Systems Inc., New York, NY, USA) that drives syringes in the size range 0.5 mL to 60 mL. The cell plate holder was additively manufactured in VeroBlack (Stratasys Ltd., Minnesota, MN, USA) material using an Objet Connex 500 (Stratasys Ltd., Minnesota, MN, USA) and mounted on a 150 × 150 mm^2^ optical breadboard with M6 screws (Thorlabs Inc., Newton, NJ, USA), raised 50 mm by mounting posts. 

The system, including translation stages, signal generator and syringe pump, was controlled by a LabVIEW-based interface (National Instruments, Newbury, UK) which allows individual wells in the cell plate to be selected, and the US and delivery parameters to be varied for each well. 

#### 4.4.4. Barrier Function Measurements during Insonation

Transepithelial resistance (TER) measurements (Equation 5) were used to assess the barrier function of cells grown on porous membranes, such as ThinCerts. Since TER is a measurement of barrier function, a reduction in value indicates a decrease in barrier function. The aim of the experiments described below was to investigate the effect of insonation on TER.

Cells were grown on ThinCerts until they reached the TER reflecting full barrier function as described in Section 4.4.1. SonoVue MBs (Bracco S.p.A., Milan, Italy) were mixed into growth medium at a concentration of 1 × 10^6^ MBs/mL. The cell plate containing the samples was then transferred into the insonation system. Nine samples were generated: three samples were exposed to MBs without insonation, three samples were insonated with no MBs present, and three samples were insonated with MBs present. A 10 *V_pp_* sinusoidal waveform was applied to the transducer continuously for 60 s per sample producing specific acoustic output parameters (last line of Table 3).

**Figure 10:**
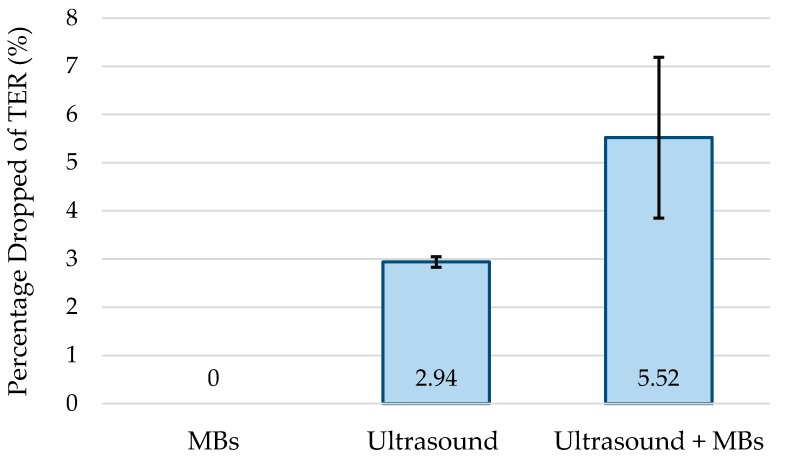
Effect of TER on small bowel model. MB-only samples were unaffected. Ultrasound-only samples had an average drop of 2.94% from initial TER. Samples exposed to ultrasound with MBs present had an average drop of 5.52% from the starting value.

Figure 10 shows the relative drop in TER in each sample type. The samples that were exposed to MBs without insonation were unaffected. Samples exposed to US alone demonstrated an average drop of 2.94% from the initial value of TER. Samples that were insonated in the presence of MBs had an average TER drop of 5.52%. In each case, the TER returned to it the initial value after 5–6 min. Since TER is a measure of the barrier function of the cell layer, a drop in TER during insonation reflects decreased barrier function. Reasons for a decrease in TER during insonation could be a release of intercellular junctions, e.g., tight junctions, or increased cell membrane permeability. Both effects could increase drug uptake. Additionally, MBs alone, which are often used as a vehicle for packaging drugs, decreased barrier function further than just US alone. This means that the miniature focused US transducers could improve uptake.

## 5. Conclusions

A potential theranostic patient pathway has been outlined (Figure 1) for the treatment of GI diseases based on the use of multimodal CE and UmTDD in a TCE. While its implementation clearly lies some way into the future, previous research and work now under way is demonstrating many important components.

US sensing in the form of qualitative and quantitative μUS analysis has been explored to diagnose diseased regions in the GI tract. Fluorescent marking is considered an important capability to allow location of diseased regions after they were identified with diagnostic CE, to allow treatment with a feasible therapeutic capsule subsequently, which will only require minimal communication or diagnostic capabilities of its own. A proof-of-concept therapeutic capsule has been constructed to determine the feasibility of UmTDD in capsule format. Viability and functional testing has shown that the miniature focused-US transducers housed in the capsule could be able to direct therapeutic agents towards a treatment site. The ability of these miniature focused-US transducers to increase permeability of a small bowel cell model and enhance therapeutic uptake has also been explored. An automated insonation system to perform drug delivery experiments was described, including specialized US transducers corresponding to those appropriate for TCE. TER, a measurement of cell barrier function, was measured across the small bowel model during insonation with MBs and a decrease was demonstrated. This indicates that the cell model is becoming more permeable and may translate to increased drug uptake in the small intestine. 

These early results demonstrate the feasibility of the proposed patient pathway. Further work is now in progress to combine diverse capabilities in single capsules, notably VCE combined with μUS imaging [6,7] for diagnosis and fluorescence imaging with UmTDD for therapy.

## Figures and Tables

**Table 1:** Specifications of commercially available capsule endoscopes [11,12,17,18,19,20,21,22,23,24].

Device	Company	Size (mm)	Weight (g)	Imaging Sensor (Pixel Res.)	Frame Rate (fps)	Angle of View (°)	Image Display	Battery Lifetime (h)
PillCam^®^ SB3	Medtronic Inc., Dublin, Ireland.	Ø11.4 × 26.2	3.0	CMOS (256 × 256)	2–6	156	Offline	8
PillCam^®^ COLON2		Ø11.6 × 32.8	3.0	CMOS × 2 (256 × 256)	4–35	172	Real Time	10
PillCam^®^ UGI		Ø11.6 × 32.8	3.0	CMOS × 2 (256 × 256)	18–35	172	Real Time	1.5
PillCam^®^ PATENCY		Ø11 × 26	3.3	N/A ^1^				
Endo-Capsule	Olympus, Tokyo, Japan	Ø11 × 26	3.8	CCD (1920 × 1080)	2	145	Real Time	8–10
OMOM System I	Jinshan Sci. & Tech., Chongqing, China	Ø11 × 25.4	≤6.0	CMOS (640 × 480)	2	140	Real Time	12
MiroCam	IntroMedic, Seoul, Korea	Ø10.8 × 24	3.3	CMOS (320 × 320)	3	150	Offline	10–12
CapsoCam Plus	CapsoVision, Saratoga, CA, USA	Ø11 × 31	4.0	CCD × 4 (221 × 884)	20	360	Offline	15

^1^ PillCam^®^ PATENCY capsule does not contain imaging components.

**Table 2:** Present status of capsules under development and their link to the proposed patient pathway described in Figure 1.

CE-Relevant Step in Patient Pathway	Required Technical Capability	Present Status of Research	Results Presented in This Paper	Relevant References
2a/4	µUS Imaging, Video Imaging, CADx	VCE established. US CE in development. Bench tests performed	Bench testing, QUS analysis	[5,6,7,80,81]
2b	US tissue marking	Proof of concept capsule developed. Tissue marking with nanoparticles demonstrated	Proof of concept capsule. Tissue marking	[9,10,82]
3a/b	UmTDD Capsule. Fluorescence Imaging	Proof of concept capsule developed and bench tested. Fluorescence imaging capsule developed; requires miniaturization.	Development of therapeutic capsule. Fluorescence capsule	[8,9,10,83,84]

**Table 3:** Output parameters of miniature focused-US transducer with central frequency *f* = 4 MHz and 1 mm central delivery channel.

Input Voltage (V_pp_)	W_input_ (mW)	W_output_ (mW)	Efficiency (%)	Pressure (kPa)	Beam Diam. (mm)	Intensity (W/cm^2^)	Mech. Index
1	2.54	N/A ^1^	N/A ^1^	11.0	2.70	N/A ^1^	0.005
2	10.2	N/A ^1^	N/A ^1^	22.9	2.70	N/A ^1^	0.011
3	22.8	8.50	37.2	34.7	2.70	0.15	0.017
4	40.6	19.9	49.0	53.7	2.70	0.35	0.026
5	63.4	34.8	54.8	61.5	2.70	0.61	0.030
6	91.4	51.0	55.8	77.4	2.70	0.89	0.038
7	124	71.3	57.4	93.3	2.70	1.25	0.046
8	162	93.6	57.6	117	2.70	1.64	0.058
9	206	123	59.8	136	2.70	2.15	0.067
10	254	153	60.3	153	2.70	2.67	0.076

^1^ W_output_ not available for low input voltages due to limited resolution of force balance.
